# Retrospective analysis of survival of avulsed and replanted permanent teeth according to 2012 or 2020 IADT Guidelines

**DOI:** 10.1590/0103-6440202305255

**Published:** 2023-05-15

**Authors:** Liliane Roskamp, Camila Paiva Perin, Juliana Pierdoná de Castro, Natanael Henrique Ribeiro Mattos, Maria Carolina Botellho Pires de Campos, Marilisa Carneiro Leão Gabardo, Sérgio Aparecido Ignácio, Maria Eduarda Nunis Locks, Vânia Portela Ditzel Westphalen, Flares Baratto-Filho

**Affiliations:** 1 Department of Dentistry. Universidade Tuituti do Paraná, Curitiba, PR, Brazil.; 2 School of Health Sciences. Universidade Positivo, Curitiba, Brazil, PR.; 3 School of Life Sciences. Pontifícia Universidade Católica do Paraná, Curitiba, PR, Brazil.; 4 Department of Dentistry. Universidade da Região de Joinville, Joinville, SC, Brazil.

**Keywords:** dental avulsion, dental replantation, guidelines, root resorption

## Abstract

This study aimed to compare the survival of replanted teeth that followed the 2012 or the 2020 International Association of Dental Traumatology (IADT) guidelines. Sixty-two permanent replanted teeth were retrospectively assessed (IADT 2012, n = 45; IADT 2020, n = 17). Five years after replantation (from January 2017 to December 2021), clinical and radiographic examinations were performed. A significance level of 95% was considered to evaluate the outcomes. Thirty-one teeth (50.0%) remained in their sockets and 31 (50.0%) were lost due to external root resorption. Of the 25 (40.3%) teeth replanted within one hour, 16 (64.0%) remained in their sockets, and 9 (36.0%) were lost. Twenty-two (71.0%) of all 31 lost teeth had an extra-alveolar time of more than one hour. Twelve teeth remained in their sockets without resorption: 8 (66.7%) were replanted within one hour, 2 (16.7%) followed the 2012 IADT, and 2 (16.7%) the 2020 IADT guidelines for late replantation. There was a significant difference (p <0.05) in the extra-alveolar time (< one hour), but without difference between the guidelines in late replantation (p > 0.05). Replanted teeth following both, 2012 or 2020 IADT guidelines, have similar clinical outcomes. The extra-alveolar time of less than one hour was demonstrated to be important to keep the permanent tooth in its socket.

## Resumo

Este estudo teve como objetivo comparar a sobrevida, por cinco anos, de dentes reimplantados que seguiram as diretrizes de 2012 ou 2020 da *International Association of Dental Traumatology* (IADT). Sessenta e dois dentes permanentes reimplantados foram avaliados retrospectivamente (IADT 2012, n = 45; IADT 2020, n = 17). Cinco anos após o reimplante, foram realizados exames clínicos e radiográficos. Foi considerado um nível de significância de 95% para avaliar os desfechos. Trinta e um dentes (50,0%) permaneceram em seus alvéolos e 31 (50,0%) foram perdidos por reabsorção radicular externa. Dos 25 (40,3%) dentes reimplantados em uma hora, 16 (64,0%) permaneceram em seus alvéolos e 9 (36,0%) foram perdidos. Vinte e dois (71,0%) de todos os 31 dentes perdidos tiveram um tempo extra-alveolar superior a uma hora. Doze dentes permaneceram em seus alvéolos sem reabsorção: 8 (66,7%) foram reimplantados em uma hora, 2 (16,7%) seguiram a IADT de 2012 e 2 (16,7%) as diretrizes da IADT de 2020 para reimplante tardio. Houve diferença significativa (p<0,05) no tempo extra-alveolar (< uma hora), mas sem diferença entre as diretrizes no reimplante tardio (p > 0,05). Dentes reimplantados seguindo as diretrizes de 2012 ou 2020 da IADT, tiveram taxas de sucesso semelhantes. O tempo extra-alveolar inferior a uma hora demonstrou ser importante para manter o dente permanente em seu alvéolo.

## Introduction

Dental avulsion is the most difficult and controversial trauma to permanent teeth. Its incidence varies from 0.5% to 16% of all traumatic injuries ^1^. Immediate replantation is the best treatment, and when it is performed properly, the tooth has a favorable outcome ^2^.

Because most cases of tooth avulsion occur in children, the greatest challenge to dentists is keeping the replanted tooth in its socket for as long as possible ^3^ ^,^ ^4^. Thus, in addition to the psychological advantage of not having a missing tooth in childhood or adolescence, the maintenance of bone quality and thickness during facial growth encourages dentists and patients to replant and follow-up the tooth ^5^.

Factors influencing prognosis include emergency care, extra-alveolar time, tooth management, replantation technique, the storage medium where the tooth was kept until replantation, time and quality of the endodontic treatment, the use of systemic medication, and follow-up ^2^ ^,^ ^6^ ^,^ ^7^. The period that an avulsed tooth is out of its socket is inversely proportional to the likelihood of success ^3^. Ideally, dental replantation should be performed within five minutes to achieve regeneration of the periodontal ligament (PDL) and to restore the tooth to normal function since viable PDL cells on the root surface of the replanted tooth are a protective factor against resorption ^8^ ^,^ ^9^.

Authors indicate that unfavorable outcomes occur mostly after late replantation, i.e., when the avulsed tooth is kept dry for more than one hour or it is not stored in a suitable medium, as milk (maximum of 6 hours), Hank´s Balanced Saline Solution - HBSS (maximum of 72 hours) until replantation ^1^ ^,^ ^2^ ^,^ ^3^ ^,^ ^7^ ^,^ ^8^, with a reported prevalence of 57% to 80% ^3^. Infection-related and ankylosis-related root resorption are the most critical consequences because they are the main causes of the extraction of the replanted tooth ^10^ ^,^ ^11^ ^,^ ^12^. However, although rare, there are indications in the literature of delayed replantations, such as an avulsed tooth with 22 ^13^ and 16 extraorally hours ^14^ that were successful.

Few authors in the literature address the molecular and cellular mechanisms involved in root resorption after trauma ^15^. Some suggest that cementum and dentin have the potential to change their cellular functions in the tooth-PDL space after an avulsion, by inducing specific and non-specific immuno-inflammatory responses. A reaction to contain the antigen can also harm the root surface, and even cause root resorption ^16^ ^,^ ^17^. The role of bacteria and modulating molecules in the PDL are also important factors in the destruction of bone and/or root surface.

Guidelines for the emergency management of dental traumatic injuries are useful for delivering the best care. The International Association of Dental Traumatology (IADT) has developed a dental avulsion guideline in 2012 ^18^, and a consensus statement after an update was published in 2020 ^8^. The IADT does not guarantee to dentists or patients favorable outcomes in following these guidelines, but believes that their application can maximize the probability of positive results.

Both guidelines report replanted teeth in less than one hour as an immediate replantation technique; more than one hour is considered a late replantation technique, regardless of if it is 61 or more minutes after the avulsion ^8^ ^,^ ^18^. From a clinical point of view, it is important for the clinician to assess the condition of the PDL cells by classifying the avulsed tooth into one of the following three groups before commencing treatment: A. When the tooth has been replanted immediately or within a very short time (about 15 minutes) at the place of the accident, the PDL cells are most likely viable. B. When the tooth has been kept in a storage medium (eg, milk, HBSS (Save-a-Tooth or similar product), saliva, or saline, and the total extra-oral dry time has been <60 minutes), the PDL cells are impaired but maybe still viable. C. When the total extra-oral dry time has been more than 60 minutes, regardless of the tooth has been stored in a medium or not, the PDL cells are probably non-viable. These three groups provide guidance to the dentist on the prognosis of the tooth ^8^.

Unfortunately, neither the community nor some dentists around the world are aware of these guidelines, and sometimes the teeth are not adequately managed in emergency care and follow-up. Therefore, this study aimed to compare the clinical outcomes after five years of replanted teeth that followed the 2012 or 2020 IADT guidelines.

## Material and methods

### Ethical aspects

Informed consent was obtained from all patients or guardians. The Local Research Ethics Committee, under the number of registration 02320084000-10, approved this research.

### Study design and sampling

In this study, a follow-up of 5 years was possible to be done with 62 teeth that were avulsed, replanted, and received clinical and radiographic evaluations. This sample included teeth of patients in good health, who didn’t suffer a posterior dental trauma or received orthodontic treatment and attended the Dental Trauma Clinic every 15 days or at least every 30 days in 12 months twice a year for five years or at least at the time of tooth extraction.

Two groups were created: teeth that were replanted and received endodontic treatment following the 2012 IADT guideline ^18^, and another group of teeth that did not follow this guideline because of a lack of information in the emergency care, but were handled, maybe by instinct (the dentists did not know any guideline), as it is now suggested by the 2020 IADT guideline ^8^. For this reason, it was possible to create a 2020 IADT guideline group.

### Intervention

*2012 IADT 2012 group*

For the formation of this group (n = 45), teeth with complete apices that were replanted with an extra-oral dry time of less than 60 minutes were held by their crowns, cleaned in running water, and replanted in their sockets. They were cleaned, shaped, and received a calcium hydroxide (CH) root filling for up to two weeks after replantation. One month after the accident, the final endodontic treatment and crown restoration were performed.

The teeth with an extra-oral dry time longer than one hour and closed apices had their PDL removed with a gauze and their root surfaces were treated with sodium fluoride for 20 minutes. Their root canals were cleaned and shaped out of the socket, and a CH paste was placed inside the root canal before replantation. The endodontic treatment was performed for up to six weeks, in the absence of signs of root resorption.

*2020 IADT group*

The 2020 IADT group (n = 17) was composed by teeth with complete apices that were replanted with an extra-oral dry time of less than 60 minutes and were held by their crowns, cleaned in milk, and replanted in their sockets. They were cleaned, shaped, and received a CH root filling for up to two weeks after replantation still with the splint in the mouth. One month after the accident, the final endodontic treatment and crown restoration were performed.

The teeth, kept dry for more than one hour, were replanted without the cleaning and shaping of the root canal before replantation. Their root surfaces weren´t treated with sodium fluoride nor was the PDL removed with gauze, but gently cleaned with milk. Their roots weren´t touched. In the next appointment, up to two weeks, these teeth were cleaned, shaped, and received CH paste as a temporary filling, with the splint in place. After at least one month, if their roots didn´t show any signs or symptoms of root resorption, the teeth received a permanent root canal filling.

All teeth with incomplete apices were followed radiographically and the pulp sensibility test was performed every 30 days to establish the status of the pulp, regardless of the extra-oral time. The teeth that responded positively to the pulp tests and/or showed continuous root development did not receive endodontic treatment. Apexification or revascularization was performed in teeth with clinical signs of infection and necrotic pulp, as well as in the presence of swelling, abscesses, and fistula, with radiographic signs of periapical lesions or indicating the interruption of root development.

All teeth, from both groups, were splinted with a flexible orthodontic wire for two weeks, and antibiotics, anti-inflammatories, and a painkiller were prescribed.

### Clinical and radiographic data

Clinical evaluations recorded the color, mobility, percussion sound, and incisal alignment that might suggest ankyloses, painful symptoms, and the presence of a fistula. The initial radiographs taken during emergency care soon after replantation were compared with those obtained after five years of the avulsion. A sensitivity E/F, size 2 Kodak Insight periapical films (Eastman Kodak Co, Rochester, NY, USA), and JON radiographic positioners (São Paulo, SP, Brazil) were employed to obtain the periapical radiographs.

All radiographs were analyzed with the aid of a 4× magnifier and dark cardstock. Two calibrated Ph.D. endodontists (more than 15 years of experience) evaluated, independently, the teeth clinically and radiographically with a very high intraexaminer agreement for both (Kappa = 0.90 and 0.88, respectively), and very good interexaminer agreement (0.88). Radiographic signs such as the presence or absence of external root resorption, complete presence, total or partial interruption of lamina dura, ankyloses, and bone loss were evaluated.

The follow-up occurred from January 2017 to December 2021.

### Statistical analysis

To evaluate the outcome of replanted teeth that followed the 2012 or 2020 IADT guidelines, the software Statistical Package for the Social Sciences (SPSS), version 23 (IBM Corp., Chicago, IL, USA) was used. The variables were: resorption (presence or absence, or tooth loss), IADT guideline (2012 or 2020), extra-alveolar time (less or more than one hour), and patients’ age at the time of replantation. To evaluate the outcome of replanted teeth, the clinical and demographic variables, this study employed the Pearson Chi-squared test, and the Z test for differences between two proportions when the sample size was small, with Bonferroni correction. The nonparametric statistical test for comparing three or more independent groups was the Kruskal-Wallis or Mann-Whitney tests for comparison between two independent groups. A significance level of 95% was considered (p < 0.05).

## Results

Of the 62 permanent teeth, 31 (50.0%) remained in their sockets, and 31 (50.0%) were lost after five years due to external root resorption. Regarding the evaluation of the presence or absence of root resorption in permanent teeth that remained in their sockets and the tooth loss of patients, whose replantation followed IADT 2012 and 2020 guidelines, no differences occurred (p = 0.322) (Table 1).

Of 25 (40.3%) teeth replanted within one hour, 16 (64.0%) remained in their sockets, and 9 (36.0%) teeth were lost after five years of replantation. Besides, 22 (71.0%) of all 31 lost teeth, had an extra-alveolar time of more than one hour. Regarding the 12 teeth that remained in their sockets without root resorption, 8 (66.7%) teeth were replanted within one hour, 2 (16.7%) teeth followed the 2012 IADT, and 2 (16.7%) followed the 2020 IADT for late replantation, without a statistical difference (p = 0.100) (Table 2). No significant difference occurred between the guidelines in late replantation, after five years of follow-up (p = 0.300) (Table 3). However, there was a significant difference (p = 0.030) regarding the extra-alveolar time (Table 4).

Of the 14 teeth that needed to be extracted due to severe root resorption, in the 2012 IADT group, 10 (71.4%) had an open apex. All of them were replanted after one hour of extra-alveolar time.

**Table 1:** Evaluation of the presence or absence of root resorption in permanent teeth that remained in their sockets and the tooth loss of patients, whose replantation followed IADT 2012 and 2020 guidelines (n = 62).

Resorption after five years	IADT 2012	IADT 2020
Absent	10a	2a
	83.3%	16.7%
	22.2%	11.8%
Present	14a	5a
	73.7%	26.3%
	31.1%	29.4%
		
Tooth loss	21a	10a
	67.7%	32.3%
	46.7%	58.8%

Note: Equal letters in the column indicate no significant difference (p > 0.05). Pearson Chi-square test (p= 0.322), and the Z test for differences between two proportions, with Bonferroni correction.

**Table 2:** Evaluation of the presence or absence of root resorption and the tooth loss in relation to the extra-alveolar time (n = 62).

Resorption after five years	IADT 2012 /less than one hour	IADT 2012 / more than one hour	IADT 2020 / more than one hour
Absent	8a	2a	2a
	66.7%	16.7%	16.7%
	32.0%	10.0%	11.8%
Present	8a	6a	5a
	42.1%	31.6%	26.3%
	32.0%	30.0%	29.4%
Tooth loss	9a	12a	10a
	29.0%	38.7%	32.3%
	36.0%	60.0%	58.8%

Note: Equal letters in the column indicate no significant difference (p > 0.05). Pearson Chi-square test (p = 0.100), and the Z test for differences between two proportions, with Bonferroni correction.

**Table 3:** Mean, standard deviation (SD), p-value and 95% confidence interval (95%CI) for the two guidelines (n = 62).

Guideline	n	Mean	SD	p-value*	95%CI	
					Lower limit	Upper limit
IADT 2012	45	1.2	0.8	0.300	1.0	1.5
IADT 2020	17	1.5	0.7		1.1	1.8

*Mann-Whitney test.

**Table 4:** Mean, standard deviation (SD), p-value and 95% confidence interval (95%CI) in relation to the extra-alveolar time (n = 62).

Guideline/Extra-alveolar time	n	Mean	SD	p-value*	95%CI	
					Lower limit	Upper limit
IADT 2012 / less than one hour	25	1.0(a)	0.8		0.7	1.4
IADT 2012 / more than one hour	20	1.5(b)	0.7		1.2	1.8
IADT 2020 / more than one hour	17	1.5(b)	0.7	0.030	1.1	1.8

*Kruskal-Wallis test. Different letters in the column indicate significant difference (p < 0.05).

## Discussion

This study indicated a 50.0% of survival rate of avulsed and replanted teeth, after five years of follow-up. Other authors ^7^ obtained the same value.

The IADT replantation guidelines are continually revised and advanced ^19^ ^,^ ^20^; however, the root resorption mechanism is still not fully understood. Therefore, this study proposes a comparison in the replantation cases that followed either 2012 or 2020 IADT guidelines. Most replanted teeth followed the 2012 IADT guideline ^18^, of which were current when the accidents happened. Cases that are now included in the 2020 IADT were managed instinctively in emergency care, and didn´t follow the 2012 IADT because the dentist didn´t know it. By coincidence, some teeth were replanted in the way the new 2020 IADT guideline suggests. For this reason, this research was possible to be developed. The resorption and loss of late replanted teeth, which correspond to the main points of change suggested by the new 2020 IADT guideline ^8^, accounted for most cases.

The treatment for replanted teeth depends on two important criteria, such as root development and viability of the PDL cells. The pulp of the tooth with an open apex can undergo spontaneous revascularization and may continue its development and maturation without endodontic treatment. The viability of the cells of the PDL is defined by the extra alveolar dry time and the storage medium of the avulsed tooth until replantation ^1^ ^,^ ^8^ ^,^ ^18^.

The extra-alveolar time is a crucial factor in maintaining the health of the PDL components of the replanted tooth ^18^ ^,^ ^21^. Exposing the root surface to an external environment and its improper handling until replantation diminishes the survival of the PDL cells. Studies have shown that after dry storage for more than 15 minutes, the existing precursor cells in the PDL may be unable to divide and differentiate into fibroblasts. After 30 minutes in a dry environment, PDL cells can die ^22^. The 2020 IADT guideline considers that the ligament cells are still viable when the tooth was kept out of its socket for up to 15 minutes. The PDL cells may be viable, but compromised when the tooth has been kept in a storage medium such as milk, HBSS, saliva, or saline, and the total extra-alveolar dry time has been less than one hour. The PDL cells are likely to be non-viable when the total extra-alveolar dry time has been more than one hour, regardless of its storage medium until replantation ^8^.

The results here presented support the importance of maintaining the tooth for less than one hour out of its socket ^18^ ^,^ ^23^. Statistically significant results were found for teeth that remained in their sockets after five years of follow-up and whose replantation was performed within one hour of extra-alveolar time. When the extra-alveolar time is less than one hour and the damage to the PDL constituents is limited, teeth have an advantage in the repair of the tooth surface/ligament space, preventing or at least delaying the damage on the root surface

In dental avulsion, it may be necessary to clean the tooth before replantation. Cleaning it in running water was previously indicated ^18^, however, some authors have indicated that water, due to its hypotonicity and low osmolarity, can lead to rapid cell lysis ^1^. For this reason, in the last guideline, milk is indicated for cleaning the root before replantation ^1^, even though running water is still acceptable. So, in this study, a unique group of less than one-hour replantation was created. In this study, the mean value of the group of replanted teeth with less than 60 minutes of extra alveolar time was smaller compared to the two other groups, when the extra-alveolar time was more than 60 minutes, regardless if they followed either the 2012 or 2020 IADT guidelines. These results demonstrate the importance of immediate replantation.

According to the 2012 guideline, the acceptable storage media were Hanks' Balanced Saline Solution (HBSS), saline, saliva, and milk ^18^. In the new update of 2020, the indicated storage media are milk and HBSS. Milk is the best option, due to its chemical and biological properties, and its capacity to maintain the viability of the cells, besides being easily obtained at the site of the accident ^8^. HBSS is used as a buffer system in cell culture media to maintain the pH between 7.0 and 7.4, which is ideal for cellular growth. Saline and saliva are still considered acceptable in the 2020 IADT guideline, even though they are options just in cases where milk is not accessible. The worst outcome occurs when the avulsed tooth is kept dry ^8^ ^,^ ^12^ ^,^ ^24^.

Sodium fluoride had been suggested to inhibit root resorption in late replantation ^1^. It probably acts by converting the hydroxyapatite present in dentin, into fluorapatite, which is more resistant to resorption ^1^. However, due to the lack of scientific evidence, the use of sodium fluoride, as well as any other substance on the root surface before replantation was discouraged in the 2020 IADT guideline ^8^. In this research, it was not found any significant difference between the groups (p > 0.05) of late replantation which teeth received the root canal treatment before replantation and fluoride in the root surface, as state by the 2012 IADT guideline ^18^ and those that followed the 2020 IADT technique ^8^, where the root surfaces are not cleaned nor the root canal treatment performed before replantation. However, it is worth noting that maybe a substance or technique that prevents external root resorption will be discovered.

The application of root canal dressing as soon as possible in teeth with closed apices may prevent damage caused by pulp death, modulate the inflammation caused by trauma, prevent infection, and minimize the risks of external root resorption ^25^. The 2020 guideline for late replantation suggests that the tooth must be replanted and splinted before the placement of a root canal dressing, in the emergency care appointment. The authors suggest that the manipulation of the root before replantation may damage its surface, increasing the possibility of the development of a resorptive process ^8^. But, if the clinical management of the patient turns out to be impossible to make the endodontic treatment in the emergency section, it is acceptable to wait for ten to14 days to do the root canal treatment.

As a limitation of this study, it is important to state that it is difficult to accomplish an *in vivo* design with children who have suffered dental avulsion. It is necessary to have a complete commitment of the patients and their guardians or close relatives for a long period, such as the five years described. It was responsible for the loss of follow-up of some of them, reducing the number of the sample. Besides, the mean values of extra-alveolar time and the clinical outcomes were not recorded. More long-term dental replantation studies are necessary to confirm the results. It is important to point out that, this study is primarily about the role of extra-oral root canal treatment and root manipulation of mature teeth that have a long extra-oral time.

In conclusion, considering the teeth in their sockets after five years of follow-up, this study indicates that replanted teeth following both 2012 and 2020 IADT guidelines for late replantation have similar clinical outcomes. The extra-alveolar time of less than one hour is important to the health of the tooth in its socket.
